# Addressing Noncommunicable Disease in Dominican Republic: Barriers to Hypertension and Diabetes Care

**DOI:** 10.29024/aogh.2370

**Published:** 2018-11-05

**Authors:** Bryan Castro, Louise Ing, Yeri Park, Jasmine Abrams, Mark Ryan

**Affiliations:** 1 Virginia Commonwealth University, US

## Abstract

**Introduction:**

Noncommunicable diseases (NCDs) significantly contribute to morbidity and mortality worldwide. During medical brigades in Santo Domingo, the Dominican Aid Society of Virginia (DASV) collects data to help understand the dynamics of NCD management. This study presents findings regarding resources and barriers to NCD treatment.

**Methods:**

A cross-sectional survey study was conducted in two communities (Los Mina and Paraiso) during the 2014 DASV summer brigade. Descriptive statistics, associations, correlations as well as qualitative analyses were conducted to better understand resources and barriers to care in relation to health care coverage.

**Results:**

More than one third (*n* = 64) of 165 individuals had hypertension and/or diabetes. Thirty-seven percent (Paraiso) and 46% (Los Mina) of study participants did not have health insurance in the previous year. For those that did have insurance, 77% (P) and 89% (LM) visited a physician in the previous year. In this same group, 65% of individuals from Paraiso reported that their health insurance never covered the cost of medications while only a quarter of individuals from Los Mina indicated this. Health insurance and access to physicians and medication varied depending on the community of residence. Surveys indicated that access to affordable medications was an important issue for participants. Also, even though individuals in Los Mina were less likely to have health insurance than those in Paraiso, they were more likely to visit a physician.

**Conclusion:**

This study contributes to a greater understanding of health care coverage and access for low-resource communities in the Dominican Republic. Health care access, insurance, and cost sharing differed between these communities, but barriers to care were common. Future investigations could focus on qualitative differences in communities' health insurance coverages and development of interventions to address obstacles to care.

## Introduction

Noncommunicable diseases (NCDs) are responsible for more than 36 million deaths worldwide, affecting all ages [1]. NCDs are non-infectious and non-transmissible chronic diseases that are divided into four categories – cardiovascular disease (CVD), diabetes (DM), cancers, and chronic respiratory diseases [1]. Forty percent of deaths due to NCDs are premature deaths before the age of 70, and the majority occur in low- and middle-income countries. NCDs were responsible for 68% of all deaths in 2012, with cardiovascular disease accounting for 52% and diabetes accounting for 6% [2]. During 2012, there was approximately a 15% chance of dying between ages 30 and 70 as a result of an NCD [3].

Both cardiovascular disease and diabetes are among the world’s leading causes of death and continue to be on the rise [4]. Reflective of international trends, rates of CVD and DM are on the rise in the Dominican Republic. Hypertension is the second leading cause of death in the Dominican Republic, while diabetes is the sixth leading cause of death. Nearly 35% of Dominicans are hypertensive and approximately 10% have diabetes [5].

In order to address the increasing burden of NCDs globally, the World Health Organization established the Global NCD Action Plan. It focuses on the integration of different sectors, including health, finance, education, and agriculture, as well as reduction of risk factors such as tobacco use and unhealthy diet. The action plan establishes the need for people-centered primary care and universal health coverage for early detection and NCD screening [1].

The Dominican Republic health system has similarly recognized the large burden that NCDs have on morbidity and mortality and has developed several initiatives [6]. The health system in the Dominican Republic includes both public and private sectors, with the Ministry of Public Health being the main provider in the public sector. Since 2001, a series of laws were passed in the Dominican Republic to promote universal health coverage, which includes preventive care and outpatient drug coverage [7]. In 2008, the Directorate for Primary Care was updated with a plan to integrate chronic disease programs into the primary health care model. The National Chronic Non-communicable Disease Control and Prevention Program was an action plan developed in 2009 by the Ministry of Public Health, aimed at prevention and management of NCDs, including associated risk factors [6].

Theoretically, such programs would enable patients to have access to a consistent physician who could help with the management of NCDs, and provide the opportunity for routine care and surveillance, lowering the chance of patients being lost to follow up. Unfortunately, due to lack of human and financial resources, this health care model has yet to be reviewed and fully implemented. Despite these efforts, inconsistencies in health care coverage remain. Globally, lack of access to health insurance is associated with worse health outcomes [8,9]. The purpose of the current study was to assess two communities’ experiences in accessing care for NCDs and what barriers might exist to such care.

## Current Study

The Dominican Aid Society of Virginia (DASV) has conducted medical brigades in greater Santo Domingo locations since 2006 (initially yearly, then twice yearly starting 2009) with the help of professional and student volunteers with backgrounds in medicine, pharmacy, nursing, and public health. Currently, the cumulative three weeks per year in-country are spent providing antiparasitic treatment for all patients, vitamins for children and pregnant women, and medical treatment for infections, cardiovascular and other chronic diseases, and, to a lesser extent, musculoskeletal symptoms. Currently, DASV operates clinics at two different sites in greater Santo Domingo. The first clinic site is set up in a school that serves residents of Los Altos del Paraíso and Esfuerzo del Paraíso of Santo Domingo Norte. The second clinic site operates in Fundación Sol Naciente, a non-profit community clinic in Los Mina, an urban section of Santo Domingo Este.

In recent years, concerns about access to and sustainability of medical services and medications, especially for individuals with chronic conditions, have prompted investigative efforts from the DASV. As indicated by Global NCD Action Plan, knowledge about community members and perceptions of chronic health conditions can assist in facilitating the development of culturally tailored initiatives to address such health issues. The growing awareness of the importance of NCDs in global health and the clinical experiences of the DASV providers led to a research project in the summer of 2013.

A qualitative study consisting of structured interviews with all known patients with hypertension and diabetes (*n* = 20) was conducted in Esfuerzo del Paraiso to elucidate knowledge and beliefs regarding the two diseases, including barriers and resources for controlling these chronic diseases within the community. Individuals highlighted the need for improved access to healthcare and medications, with a special focus on issues of finances and transportation/location of resources (Jasmine Abrams, Bryan Castro, Sushmita Gordhandas, Anna Grzegorczyk, and Mark Ryan, manuscript submitted). The current study is a follow up to the previously described work. In the current study, a larger population was surveyed about recurring themes identified in the qualitative study surrounding resources and barriers to care in relation to their health care coverage. An understanding of context and generalized community beliefs may help determine implications for future clinical care for residents of Santo Domingo and elsewhere in the Dominican Republic. These issues may also be more broadly applicable to other nations.

## Methods

This research study was approved by the VCU IRB under protocol HM20002559. Based on the results from the previously mentioned qualitative study, a questionnaire (Table 1) was developed to explore the access of these communities to medical care and medications. After administering only four surveys, question 3 was found to contain two separate characteristics (i.e., frequency of health insurance coverage for physician visits versus frequency of health insurance coverage for medications) and henceforth two distinct responses were collected.

**Table 1:** Survery Questions Assessing Access to Medical Care and Medications.


1.	In the last 12 months, have you been diagnosed with hypertension, diabetes, neither or both?
2.	In the last 12 months, have you had health insurance?
3.	A: In the last 12 months, if you had health insurance, did it cover the costs to see the doctor? B: In the last 12 months, if you had health insurance, did it cover the costs for medications?
4.	In the last 12 months, have you visited a doctor?
5.	In the last 12 months, have you needed to go to the doctor but haven’t been able to?
6.	In the last 12 months, have you had difficulty obtaining medications?
7.	If you unable to go to the doctor, what was your primary reason?
8.	What are the top three reasons that delay or prevent you from seeing your doctor or obtaining medications?


A cross-sectional design was utilized. The surveys were administered at both Paraiso and Los Mina during the summer brigade of 2014. Systematic sampling was used to identify every fifth patient leaving the clinic as a potential participant in the study. If the identified patient was under the age of 18, an accompanying adult was asked to participate instead. All participants gave verbal informed consent before participation. Individuals were offered the survey to complete or were offered assistance to complete the survey, but not one individual opted for the former. Survey questions were asked in Spanish, and the surveys were completed on paper after patients had received all available medical care. Participants were not compensated for their participation.

Data entry was completed by three trained researchers and reviewed once more by a fourth party for accuracy and quality control. Descriptive statistics were calculated for each of the characteristics assessed. Associations between the two locations, health insurance status, and receiving medical attention in the last 12 months were calculated by computing odds ratios. Kendall Tau-b correlation analyses were conducted for ordinal variables: frequency of health insurance coverage for physician visits and medicines, frequency of needing to see a physician but not being able to, frequency in obtaining medications. All quantitative analyses were conducted using SPSS 22 and qualitative analyses were conducted by coding answers to free response questions and coalescing codes into themes.

## Results

**Figure 1:**
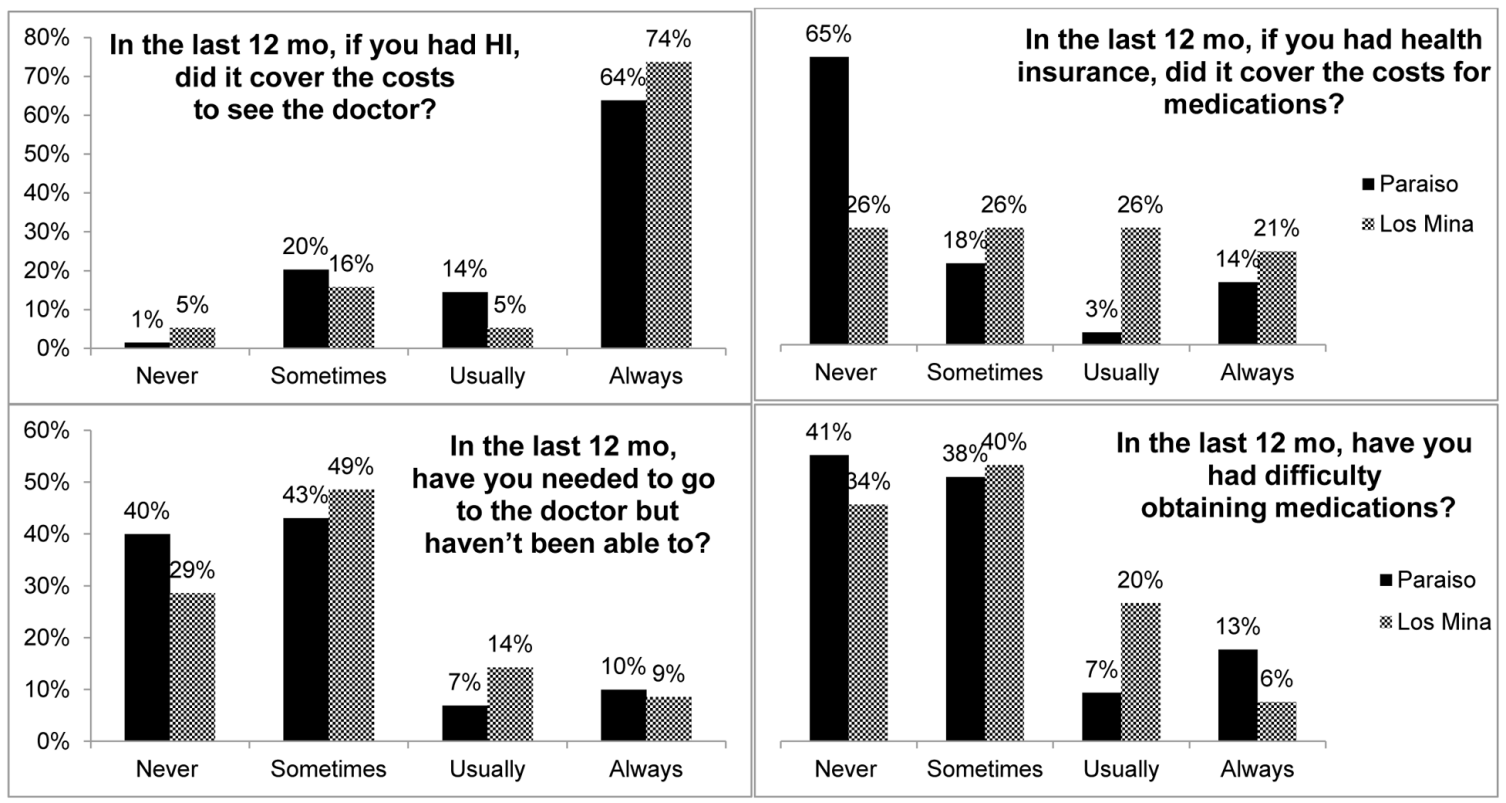
Health insurance coverage for physician visits and medication, and difficulty in seeing a physician and obtaining medication.

Of the 173 individuals who were approached, 165 agreed to participate and were included in the current study. The final sample included 130 participants from Paraiso (P) and 35 from Los Mina (LM), a participation rate of 98% and 82% for these locations in that order. General descriptive statistics can be found in Table 2. Proportions differ for both locations, but more than one third of the individuals had hypertension, diabetes, or both, more than half had health insurance in the last 12 months, and more than three-fourths visited a physician in the last 12 months. Figure 1 shows the frequency of health insurance coverage for physician visits and medications and also the difficulty in seeing a physician or in obtaining medications.

**Table 2:** Descriptive Statistics.

Health Condition	Paraiso n (%)	Los Mina n (%)

Hypertension	38 (29.7)	10 (28.6)
Diabetes	6 (4.7)	0 (0)
Both	8 (6.3)	2 (5.7)
None	76 (59.4)	23 (65.7)
**Health Insurance**	86 (63.2)	19 (54.3)
**Doctor Visit**	100 (76.9)	31 (88.6)

For those with health insurance, 64% (P) and 74% (LM) indicated that their health insurance covered physician visits, and 83% (P) and 70% (LM) never or sometimes had difficulty seeing a physician. In this same group, 65% of individuals from Paraiso reported that their health insurance never covered the cost of medications while only a quarter of individuals from Los Mina indicated this; 79% (P) and 74% (LM) never or sometimes had difficulty obtaining medication. Individuals with health insurance were 2.78 times as likely to go to the doctor (OR CI: 1.29, 6.01). Individuals in Los Mina were 2.3 times as likely to visit a doctor as those in Paraiso (OR CI: 1.76, 7.1). Individuals who live in Los Mina were 39% less likely than those in Paraiso to have health insurance (OR CI: 0.28, 1.3). There was also a significant negative correlation between difficulty obtaining medications and whether or not health insurance includes the cost of medications (*r* = –0.269, *p* = 0.004).

## Discussion

This cross-sectional study extends previous findings of the initial qualitative project, indicating that common barriers prevent Santo Domingo community members from accessing health care and resources. In this study, a larger population was sampled from Paraiso, as well as from the community of Los Mina. Results revealed that individuals with health insurance were more likely to visit physicians. Interestingly, those who live in Los Mina with less insurance coverage were more likely to use medical services, and individuals from Paraiso with more insurance coverage were less likely to use medical services. This could be explained by the locations of the clinical settings where surveys were collected. In Los Mina, surveys were collected in a low-cost clinic whose focus is on providing care for patients with difficult access to healthcare, and this could have led to an over-representation of uninsured patients who came to this clinic for their care. Meanwhile, the community of Paraiso is approximately 30–45 minutes away (by foot) from local government health facilities, which may limit ease of access to the clinical location. However, patients in Paraiso with health insurance may be better able to access care at those locations once they arrive.

Still, it would be of importance to investigate differences in insurance types between the two communities. The type of insurance could dictate services and amount of the various types of health care services that are covered (e.g., physician visit, hospital stay, medications, procedures, and therapy sessions). It is possible that in Los Mina, fewer people are insured, but those with insurance have greater coverage, allowing for greater utilization of medical services. The survey data suggests that access to a physician and/or healthcare center does not appear to be a limiting factor in addressing the long-term health risks associated with NCDs. Between two-thirds and three-fourths of patients reported that insurance did help pay for physicians’ visits and that there were few barriers to medical visits.

Instead, affordable and reliable access to medications may be the limiting factor. Results of this study also revealed a significant correlation between difficulty in obtaining medications and whether or not health insurance covers the cost of medications, such that as the level of medication coverage decreased, community members found it more difficult to access medications. Further, in Paraiso, 83% of patients reported that insurance never or rarely paid for medications, while in Los Mina, 52% of patients reported the same. This difference might be explained by which insurance is most likely found in each community, but in either case, no more than half of patients found that their medications were reliably paid for by insurance. This relates to the finding that 20–25% of residents in each community noted that they usually or always struggled to obtain medications. These data indicate that steady access to needed medications is a potential gap in the Dominican Republic’s plans to address NCDs and that focusing on this issue may have a rapid and meaningful impact in reducing the harms of NCDs.

This study is important for clinicians and organizations providing care in low-resource communities, especially in developing nations. As NCDs increasingly become a focus of medical care in low- and middle-income nations, understanding the barriers to care is a key factor in understanding how to provide direct care in the clinic as well as how to work at the organizational level to partner with communities in order to overcome these barriers. The study is also important when considering the role of public health systems in addressing NCDs. In the Dominican Republic, primary care is organized around communities and geographic areas, and public health centers (primary health centers, or centros de primer nivel) are responsible for tracking and treating NCDs in their populations. This study provides an assessment of challenges these centers may face and may help support steps to increase access to both medical care and to needed medications.

Finally, the survey in this study was easily deployed in a high-volume medical outreach clinic, and would be easily reproduced or adapted in other settings. Any organization providing health care services must attempt to understand the context in which this care is being provided. Short-term medical trips are not freed from this responsibility, but the nature of these trips may create challenges in developing rapid and useful assessments of the NCD and health insurance environment of the community being served. We believe our survey may be a useful starting point for others.

## Limitations

Despite its strengths and new knowledge elicited, our study contains several limitations. One is the use of a survey that was not validated. Further, question 3 was split into two different components addressing health care access and medication but question 8 combined both into one question. In addition, given the nature of the administration of the survey (read by the administrator), a social desirability bias may have been present, which may have influenced the answers provided by participants. Lastly, there was an unequal representation of the two communities in our sample.

## Future Research

Given that a community with less health insurance was more likely to use medical services, while those with more health insurance were less likely to use medical services, it would be worth investigating the underlying reasons for this counterintuitive finding. Perhaps efforts should be targeted at evaluating reasons for decreased utilization of medical services in the setting of health insurance, which may include lack of access (i.e., lack of transportation or proximity of facilities), differences in medication coverage through health insurance, and the quality of health care. After identification of these barriers, one can then work towards improving the management of NCDs and increasing access to health care in these two communities in Santo Domingo.

## Conclusion

This study contributes to a greater understanding of health care coverage and access in low-income marginalized communities in the Dominican Republic. This information sheds light on common barriers to care experienced by low-resource communities. Knowledge of these perceptions can facilitate the development of culturally tailored initiatives to address such issues. An important finding of the current study was that access to physicians and medical centers did not appear to be a major limitation in treating NCDs. Rather, as suggested by our results, reliable access to affordable medications is a key barrier to NCD treatment.
